# Repeat Revascularization Post Coronary Artery Bypass Grafting: Comparing Minimally Invasive and Traditional Sternotomy Techniques in 1468 Cases

**DOI:** 10.7759/cureus.25687

**Published:** 2022-06-06

**Authors:** Peter Olson, Michael Cinelli, Hamfreth S Rahming, Thomas Vazzana, Jonathan Spagnola, Emad Barsoum, Marc Assaad, Frank Tamburrino, James Lafferty

**Affiliations:** 1 Internal Medicine, Swedish Cancer Institute, Seattle, USA; 2 Cardiology, Staten Island University Hospital, Staten Island, USA; 3 Internal Medicine, Staten Island University Hospital, Staten Island, USA

**Keywords:** cardiothoracic surgery, coronary artery bypass grafting, coronary revascularization, sternotomy, angiograpghy

## Abstract

Background: Traditional open sternotomy coronary artery bypass grafting (CABG) utilizes highly invasive techniques that lead to several serious complications. In response, minimally invasive cardiac surgery CABG (MICS-CABG) was developed. MICS-CABG is safe, reproducible, and with fewer complications, while allowing for better postoperative recovery periods. There is a paucity of data exploring rates of repeat revascularization in patients post MICS-CABG compared to post traditional sternotomy CABG.

Methods: This was a retrospective billing database review examining 1468 CABG patients at a large university medical center from January 2005 to December 2017. The primary objective was to compare the rate of repeat revascularization events between MICS-CABG and traditional open sternotomy CABG over an eight-year follow-up period.

Results: Our study population consisted of 1468 patients, of whom 513 had MICS-CABG and 955 had traditional CABG. The number of patients undergoing repeat revascularization within the eight-year surveillance was 99 for MICS-CABG and 75 for traditional CABG. The Kaplan-Meier survival probability estimates for eight years were 0.86 for MICS-CABG and 0.91 for traditional CABG. The mean time until a repeat revascularization event was 84.1 months for MICS-CABG and 88.5 months for traditional CABG.

Conclusions: Traditional CABG was found to have a statistically significantly longer time to repeat revascularization than MICS-CABG. Despite the technical challenges associated with MICS-CABG, the time to repeat revascularization was different by only about four months, which may not hold large clinical significance. This suggests that MICS-CABG may have a role to play due to previous findings showing a reduction in complications while allowing for better postoperative recovery periods.

## Introduction

Coronary artery disease (CAD) has consistently been cited as the leading cause of death worldwide, unmatched by any other condition. In response to this knowledge, the international community has developed coronary angioplasty as the gold standard for revascularizing single or multi-vessel CAD, with this procedure being performed routinely around the globe [1]. However, for certain patients, mainly those who have triple vessel disease, literature has shown that the most efficient and effective way to approach myocardial revascularization is via coronary artery bypass grafting (CABG) [1-4]. Historically, CABG has been completed using a traditional sternotomy technique.

The main advantage of CABG over coronary angioplasty is total vessel replacement, as opposed to single segment correction. This reduces large lesions, while also eliminating lesions that cannot adequately be managed with angioplasty [1-4]. CABG also offers improved long-term viability of revascularized vessels as opposed to angiography [5-7]. Unfortunately, the traditional CABG operation utilizes highly invasive techniques such as open sternotomy, cardiopulmonary bypass, and induced cardiac arrest. Patient outcomes, including major morbidity, atrial fibrillation, decreased physical function, and residual pain, highlight the need for a better procedure to accomplish multi-vessel revascularization in appropriate patient populations [1,8-15].

Recent strides have been made to fill this gap with the development of minimally invasive cardiac surgery CABG (MICS-CABG). Done with the McGinn technique, this procedure allows CABG to be performed through several small chest wall incisions, rather than with an open median sternotomy. The technique also allows the procedure to be done with a beating heart, rather than with induced cardiac arrest and cardiopulmonary bypass. Literature has shown MICS-CABG to be safe as well as reproducible while allowing for better postoperative recovery periods [8-10,16,17]. In comparison to traditional sternotomy CABG, MICS-CABG has also been shown to reduce both transfusions and infections [8,10]. Further research indicates that MICS-CABG has outcomes and graft patency at least equal to traditional CABG, and superior to coronary angioplasty [17-19]. When comparing patients who have had coronary angioplasty and traditional CABG, literature shows that patients who have had CABG also have lower rates of repeat revascularization with coronary angioplasty in the future [20,21].

Traditional sternotomy CABG is the preferred approach for the revascularizing of multi-vessel CAD or in certain indications, with better long-term vessel viability than angioplasty [1]. The benefits of the procedure are partially mitigated by its highly invasive nature and subsequent complications. In response, MICS-CABG was developed in 2005. Research shows MICS-CABG to be safe, reproducible, and to have lower complication rates than traditional sternotomy CABG. MICS-CABG has also been shown to be at least equal to CABG, and superior to angioplasty with regards to outcomes and graft patency. Given the recent development of MICS-CABG, the literature has yet to show how MICS-CABG compares to traditional sternotomy CABG with respect to repeat revascularization events.

In our study, we fill the literature gap by exploring the rates of repeat revascularization events post MICS-CABG in comparison to post traditional sternotomy CABG. The study will also examine the time to repeat the revascularization event, with respect to the original surgery date. The study will help inform surgeons about the best procedure to perform by giving them hard data about the long-term efficacy of MICS-CABG in comparison to traditional sternotomy CABG.

## Materials and methods

This is a retrospective billing database review project examining electronic medical records of CABG patients at a large university medical center from January 2005 to December 2017. The primary objective of the study is to compare the rate of repeat revascularization events on one diseased vessel between the MICS-CABG group and the traditional open sternotomy CABG group over an eight-year follow-up period.

A total sample size of 1468 patients (513 patients in the MICS-CABG group and 955 patients in the traditional open sternotomy CABG group) was included in this analysis. Preoperative risk stratification was done based on the Society of Thoracic Surgeons (STS) score. Low-risk patients have an STS score of less than 4%; however, intermediate-risk patients have an STS risk of 4-8%, and 8% and above is for high-risk patients. Inclusion criteria were patients with a history of CAD that required CABG in Staten Island University Hospital from January 2005 until December 2017, patients both male and female, and aged 18 years and older. Exclusion criteria were patients with concomitant valve surgery, active or chronic inflammatory disease, active autoimmune diseases, hematological proliferative diseases, revascularization occurring on more than one vessel, and missing data on medical records.

Follow-up data of repeat revascularization events (repeat CABG or percutaneous coronary intervention (PCI)) were queried via New York State billing database for the International Classification of Diseases, Ninth Revision (ICD-9) codes of V45.81 and V45.82 and International Classification of Diseases, Tenth Revision (ICD-10) codes of Z95.1 and Z98.6 until December 2017. For patients with multiple CABG procedures, data were captured and recorded at the time of the first procedure. Subsequent procedures were documented to be repeated revascularization events. On the occasion that data were missing from this database query, investigators inquired into medical records for additional data. Patient-specific data were stored on a password-protected network file. At the earliest possible time, the patient-specific data were destroyed and entered onto the REDCap database (Vanderbilt University, Nashville, TN) as de-identified data.

All data obtained were used specifically for research purposes. Further demographic data including age, race, gender, comorbidities (diabetes mellitus, hypertension, hyperlipidemia, congestive heart failure, end-stage renal disease), type of bypass grafts, site of graft sites, and PCI site after surgery were also collected. All research was conducted at Staten Island University Hospital and approved by the institutional review board.

Summary statistics were reported as mean ± standard deviation or median (first quartile, third quartile) for continuous variables. For categorical variables, summary statistics were reported as frequency and percentages. Differences between groups in normally distributed continuous variables were analyzed with an independent sample t-test. Comparisons of skewed data were performed using non-parametric Mann-Whitney and Kruskal-Wallis methods. The chi-square or Fisher's exact test was used to compare categorical variables between groups and as a test of heterogeneity among multiple proportions.

The time to the first revascularization during the eight-year follow-up period was analyzed using survival analysis methods. The revascularization rates for the PCI and CABG groups at the end of the eight years of follow-up were estimated by the Kaplan-Meier method, with comparisons made using the log-rank test. Additionally, a multivariate Cox regression analysis that included dyslipidemia, PCI, myocardial infarction, congestive heart failure, number of diseased vessels, and surgery type (PCI or CABG) was performed to adjust for potential confounders. All analyses were two-sided and a p-value < 0.05 was considered to indicate statistical significance. All statistical analyses were performed using the SAS software, version 9.3 (SAS Inc., Cary, NC).

## Results

A total of 1468 patients completed the study. Table 1 shows the baseline characteristics of the two patient groups. The number of patients who underwent MICS-CABG was 513, and none of them converted to traditional CABG. The number of patients who underwent CABG was 955. Significant results include that the mean age was 64.8 years in the traditional CABG group and 63.4 years in the MICS-CABG group (p = 0.02). The presence of dyslipidemia was 496 in the traditional CABG group and 386 in the MICS-CABG group (p < 0.01). The presence of peripheral artery disease was 124 in the traditional CABG group and 43 in the MICS-CABG group (p = 0.01). The history of myocardial infarction was 501 in the traditional CABG group and 198 in the MICS-CABG group (p < 0.01). The history of PCI was 247 in the traditional CABG group and 93 in the MICS-CABG group (p < 0.01). The presence of congestive heart failure was 155 in the traditional CABG group and 42 in the MICS-CABG group (p < 0.01). The history of cerebral vascular accident (CVA) was 119 in the traditional CABG group and 40 in the MICS-CABG group (p = 0.01). The presence of diabetes mellitus was 354 in the traditional CABG group and 156 in the MICS-CABG group (p = 0.01). The presence of left main coronary disease was 324 in the traditional CABG group and 152 in the MICS-CABG group (p = 0.01). The left ventricular ejection fraction (LVEF) at the initial CABG event was 39.4 in the traditional CABG group and 41.4 in the MICS-CABG group (p < 0.01).

**Table 1:** Baseline characteristics CVA: cardiovascular accident; LVEF: left ventricular ejection fraction; CABG: coronary artery bypass graft; BMI: body mass index; PCI: percutaneous coronary intervention; COPD: chronic obstructive pulmonary disease; MICS: minimally invasive cardiac surgery.

Characteristic	Traditional open sternotomy CABG (N = 955)	MICS-CABG (N = 513)	P-value
Age	64.8	63.4	0.02
Gender (male)	683	384	0.18
BMI	29.5	29	0.08
Race (Caucasian)	850 (58%)	461 (31%)	0.66
Smoker	469 (32%)	254 (17%)	0.91
Hypertension	713 (48%)	404 (27%)	0.08
Dyslipidemia	496 (33%)	386 (26%)	<0.01
Peripheral artery disease	124 (8.4%)	43 (2.9%)	0.01
End-stage renal disease	23 (1.5%)	12 (0.8%)	1
History of myocardial infarction	501 (34%)	198 (13.5%)	<0.01
History of PCI	247 (17%)	93 (6.3%)	<0.01
Congestive heart failure	155 (10.5%)	42 (2.8%)	<0.01
History of CVA	119 (8.1%)	40 (2.7%)	0.01
Diabetes mellitus	354 (24%)	156 (10.6%)	0.01
COPD	121 (8.2%)	60 (4.1%)	0.62
Left main coronary artery disease	324 (22%)	152 (10.3%)	0.01
Initial CABG LVEF %	39.4%	41.4%	<0.01
Repeat Revascularization LVEF%	49.8 %	50.8 %	0.53

The primary endpoint for this study is time to repeat the revascularization event for one diseased vessel to insure the homogeneity of our sample. The estimated mean time until a repeat revascularization event was 84.1 months for the MICS-CABG group and 88.5 months for the traditional open sternotomy CABG group (Table 2). There was a significant difference in survival times between the treatment groups (log-rank test, p = 0.006). Because the survival curve for the traditional open sternotomy CABG group is above that for the MICS-CABG group, it can be concluded that the traditional open sternotomy CABG group had significantly longer times until repeat revascularization event as a whole. Therefore, the time until the repeat revascularization event was longer in the traditional open sternotomy CABG group than in comparison to the MICS-CABG group. The Kaplan-Meier survival probability estimates for eight years (97 months) were 0.86 for the MICS-CABG group and 0.91 for the traditional open sternotomy CABG group (Table 2). Cox multivariate regression completed on the data showed that after controlling for confounding factors, the hazard for the traditional open sternotomy CABG group is 0.67 times less than that of the MICS-CABG group.

**Table 2:** Repeat revascularization event time and survival CABG: coronary artery bypass graft; MICS: minimally invasive cardiac surgery; SD: standard deviation.

	Traditional open sternotomy CABG	MICS-CABG	P-value
Number of patients undergoing repeat revascularization of one diseased vessel at 8 years	99 (6.7%)	75 (5.1%)	0.02
Mean time until repeat revascularization event (months)	88.5 (SD: 1.27)	84.1 (SD: 0.94)	0.01
Probability of surviving to 8-year follow-up without repeat event	91%	86%	

## Discussion

As the SYNTAX and FREEDOM trials have demonstrated, CABG is the safest and most effective way to achieve myocardial revascularization in patients with triple vessel CAD. Although traditional open sternotomy CABG is a safe and effective option, its invasiveness using methods such as median sternotomy, cardiopulmonary bypass, aortic cross-clamping, and cardioplegia to induce cardiac arrest raises serious concerns about the physical and psychological trauma patients continue to face [17]. MICS-CABG is now a widely accepted and safer option for patients, as it has proven to have lower rates of blood transfusion, a lower incidence of chest wall wound infections, and an improved postoperative physical recovery. However, the graft patency results of MICS-CABG had not been widely studied before. Our study explores the rates of repeat revascularization in patients post MICS-CABG compared to post traditional open sternotomy CABG.

The principal finding of this study is that the average mean time until a repeat revascularization event in the MICS-CABG group was 84.1 months, compared to 88.5 months for the traditional open sternotomy CABG group. While the time interval between the two groups (4.4 months) is statistically significant, we query whether it is clinically significant in a meaningful way. The question to bear in mind is as follows: Are the invasive aspects of traditional CABG (open sternotomy, cardiopulmonary bypass, aortic clamping), increased chest wound infections, and lower post-operative physical recovery worth the cost to the patient when revascularization rates are at relatively similar timelines? In addition, MICS-CABG has shown a lower incidence of perioperative morbidity and mortality, as well as high procedural success with regard to applicability and revascularization completeness [8]. Other possible benefits of MICS-CABG include a reduction in postoperative atrial fibrillation, reduced length of hospital stay, earlier mobilization of patients, and cost-effectiveness when compared to traditional CABG [9]. When compared to traditional open sternotomy, MICS-CABG does not involve any significant infrastructure acquisition or operational costs, thereby potentially facilitating the implementation of this modality in new centers [8].

However, it should be noted that surgical selection bias in our patient population may have favored the inclusion of patients more likely to benefit from MICS-CABG. Thus, potentially leading to partially skewed results. MICS-CABG may also have its limitations, precluding widespread acceptance and use. Possible difficulties with MICS-CABG include inadequacy of heart exposure leading to longer operation times and perioperative complications, the requirement of a highly technically skilled surgeon, and possibly more pain due to involvement of the intercostal nerves and rib retraction [9]. Finally, our studied sample was not stratified according to the STS risk scoring system, which might also be a potential confounding and might affect the generalizability of our findings.

## Conclusions

Taking into account the aforementioned limitations, our study demonstrates a statistically significant result with regard to time from CABG to repeat revascularization events. We posit that, due to the large number of side effects and complications associated with traditional open sternotomy CABG, our results do not demonstrate a clinically significant finding. Given this statement, we believe that MICS-CABG has a broader role to play in the national and international community with regard to coronary revascularization of patients with CAD. We also call for a randomized clinical trial to be performed to determine head-to-head safety, patency, and survival between the two procedures.
